# Supplementary material for: A QSAR Study for Antileishmanial 2-Phenyl-2,3-dihydrobenzofurans
Source: Molecules. 2023 Apr 12;28(8):3399. doi: 10.3390/molecules28083399 (PMC10144340; doi:10.3390/molecules28083399)
Supplement: Supplementary file 1 [file molecules-28-03399-s001.zip › molecules-2301022-supplementary.pdf]

A QSAR Study for Antileishmanial 2-Phenyl-2,3-dihydrobenzofurans †

Freddy A. Bernal ‡ and Thomas J. Schmidt *

University of Münster, Institute of Pharmaceutical Biology and Phytochemistry (IPBP), PharmaCampus—Correnstraße 48, 48149 Münster, Germany; freddy.bernal@leibniz-hki.de
* Correspondence: thomschm@uni-muenster.de
† This work is cordially dedicated to the memory of Prof. A. Wilhelm Alfermann, Düsseldorf, deceased on 23 February 2023, who spent a great part of his scientific life working on natural lignans.
‡ Current address: Transfer Group Anti-Infectives, Leibniz Institute for Natural Product Research and Infection Biology, HKI, Beutenbergstraße 11a, 07745 Jena, Germany.

Contents

Table S1. Antileishmanial activity and chemical structure of compounds from class **A**. 2
Table S2. Antileishmanial activity and chemical structure of compounds from class **B**. 3
Table S3. Feature selection by contingency analysis in MOE. 5
Table S4. Equations describing models MLR1-MLR3. 11
Table S5. Model performance assessment using different validation metrics. 12
Table S6. Definition of validation parameters used for assessing the performance of the models. 13
Table S7. Definition of consensus scoring functions. 15
Figure S1. Comparison of models' performance using different metrics and PCA. 16
Table S8. Activity predictions by model 3D4. 17
Table S9. Molecular descriptors considered for model building. 18

Table S1. Chemical structure and antileishmanial activity and of compounds of class **A** *.

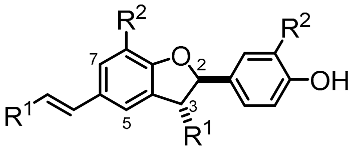

Compound	R^1	R^2	IC_{50} (μM)
**1**	$COOCH_3$	H	7.84
**2**	$COOCH_2CH_3$	H	7.45
**3**	$COOCH(CH_3)_2$	H	4.21
**4**	$COO(CH_2)_3CH_3$	H	10.8
**5**	$COOC(CH_3)_3$	H	6.07
**6**	$COOCH_3$	OCH_3	10.6
**7**	$COOCH_2CH_3$	OCH_3	9.10
**8**	$COOCH(CH_3)_2$	OCH_3	3.95
**9**	$COO(CH_2)_3CH_3$	OCH_3	3.11
**10**	$COOC(CH_3)_3$	OCH_3	2.10
**11**	$COOCH_3$	OH	12.6
**12**	$COOCH_2CH_3$	OH	3.87
**13**	$COOCH(CH_3)_2$	OH	0.54
**14**	$COO(CH_2)_3CH_3$	OH	1.66
**15**	$COOC(CH_3)_3$	OH	1.66
**16**	$COO(CH_2)_7CH_3$	OH	23.5
**17**	$CH_2CH{=}CH_2$	OH	6.32
**18**	$COOC(CH_3)_3$	F	1.97
**19**	CH_3	OCH_3	6.45
**20**	CH_2OH	OCH_3	160
**21**	$CONH(CH_2)_2CH_3$	OCH_3	8.88
**22**	$CONHCH(CH_3)_2$	OCH_3	53.6
**23**	$CONHCH(CH_3)_2$	OH	82.3
**24**	$CONH(CH_2)_2OH$	OCH_3	212
**25**	$COCH_2CH(CH_3)_2$	OCH_3	3.81
**26**	$COCH_2CH(CH_3)_2$	OH	4.42
**27**	$COCH_2C(CH_3)_3$	OH	3.42
**28**	COOH	OCH_3	6.89

* Data taken from [26].

Table S2. Chemical structure and antileishmanial activity of compounds of class **B** *.

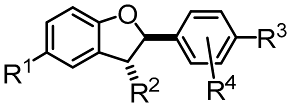

Compound	R^1	R^2	R^3	R^4	IC_{50} (μM)
**29**	H	CH_3	OH	3-OCH_3	59.0
**30**	H	CH_2OH	OH	3-OCH_3	185
**31**	H	CH_3	OCH_3	-	99.9
**32**	H	CH_2OH	OCH_3	3-OCH_3	136
**33**	H	CH_2OH	OH	-	158
**34**	H	CH_2OH	OCH_3	-	70.3
**35**	H	CH_2OAc	OCH_3	-	58.4
**36**	H	CH_2OH	-	2-Br	21.7
**37**	CHO	CH_3	OCH_3	-	30.5
**38**	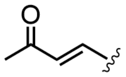	CH_3	OCH_3	-	17.4
**39**	Br	CH_2OH	OH	-	18.8
**40**	Br	CH_2OAc	OH	-	13.7
**41**	Br	CH_3	OH	3-OCH_3	24.8
**42**	Br	CH_2OH	OH	3-OCH_3	30.5
**43**	Br	CH_3	OCH_3	-	31.8
**44**	Br	CH_2OH	OCH_3	3-OCH_3	33.8
**45**	Cl	CH_3	OH	3-OCH_3	16.1
**46**	Br	CH_3	OH	2-Br, 3-OCH_3	15.0
**47**	Br	CH_2OH	OH	2-Br, 3-OCH_3	10.7
**48**	Br	CH_3	OCH_3	3-Br	29.7
**49**	Br	CH_2OH	OCH_3	2-Br, 3-OCH_3	11.4
**50**	Cl	CH_3	OH	3-Cl, 3-OCH_3	13.4
**51**	Br	CH_2OH	OH	3-Br	21.8
**52**	Br	CH_3	OH	3-OCH_3	17.2
**53**	Br	CH_2OH	OH	3-OCH_3	12.1
**54**	H	CH_2OH	OH	3,5-diBr	10.1
**55**	H	CH_2OH	OH	3-Br	43.8
**56**	H	CH_3	OH	3-I, 5-OCH_3	13.4
**57**	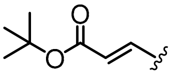	CH_3	OCH_3	-	4.01
**58**	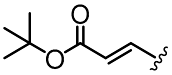	CH_2OH	OCH_3	3-OCH_3	10.7
**59**	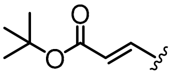	CH_2OH	OH	-	17.1
**60**	Br	CH_2OH	OH	3-OH	17.8
**61**	Br	CH_3	OH	-	32.5

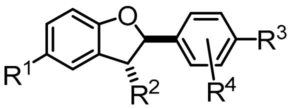

Compound	R¹	R²	R³	R⁴	IC_{50} (μM)
**62**	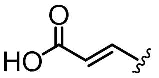	CH_3	OH	-	5.64
**63**	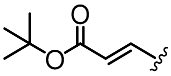	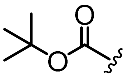	OAc	-	2.06
**64**	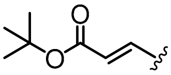	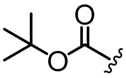	$OC(CH_3)_3$	-	13.1
**65**	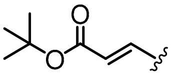	CH_2F	OCH_3	3-OCH_3	13.0
**66**	H	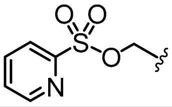	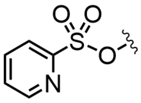	-	6.31
**67**	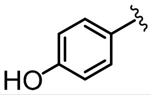	CH_2OH	OH	-	16.5
**68**	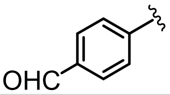	CH_2OH	OH	-	37.7
**69**	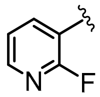	CH_2OH	OH	-	18.8
**70**	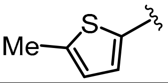	CH_2OH	OH	-	16.1

* Data taken from [26].

Table S3. Feature selection by contingency analysis in MOE.

#	Contingency coeff.	Cramér's V	Entropic Uncertainty	Linear R^2	Descriptor	Decision
2	0.81328	0.40348	0.38035	0.30404	AM1_E	Yes
3	0.79824	0.38256	0.34126	0.25895	AM1_Eele	Yes
4	0.79003	0.372	0.34153	0.26312	AM1_HF	Yes
5	0.69529	0.27926	0.21752	0.03011	AM1_HOMO	No
6	0.71633	0.29635	0.21971	0.03011	AM1_IP	No
7	0.79392	0.37694	0.35629	0.37622	AM1_LUMO	Yes
8	0.77034	0.34875	0.33147	0.26152	apol	Yes
9	0.76777	0.34592	0.32748	0.31618	ASA	Yes
12	0.76777	0.34592	0.32748	0.31618	ASA_H	Yes
16	0.71398	0.29437	0.22812	0.26918	ast_violation	Yes
17	0.78098	0.36098	0.32977	0.26624	ast_violation_ext	Yes
18	0.70396	0.28613	0.24942	0.10166	a_acc	No
22	0.908	0.28216	0.51381	0.23133	a_count	Yes
24	0.7692	0.33385	0.29296	0.03348	a_donacc	No
25	0.83202	0.29414	0.39592	0.28372	a_heavy	Yes
26	0.8292	0.34033	0.40469	0.26452	a_hyd	Yes
27	0.78252	0.3628	0.35866	0.23241	a_IC	Yes
28	0.71878	0.29845	0.26886	0.00033	a_ICM	No
31	0.8096	0.30098	0.37079	0.26351	a_nC	Yes
34	0.84457	0.26289	0.42841	0.17837	a_nH	No
37	0.72574	0.30453	0.28158	0.26038	a_nO	Yes
41	0.74084	0.3184	0.30476	0.05245	BCUT_PEOE_0	No
42	0.77105	0.34955	0.32271	0.19028	BCUT_PEOE_1	No
43	0.76336	0.34113	0.28805	0.16175	BCUT_PEOE_2	No
44	0.74175	0.31926	0.25288	0.07257	BCUT_PEOE_3	No
45	0.78868	0.37032	0.35583	0.01235	BCUT_SLOGP_0	No
46	0.77863	0.35822	0.30124	0.20128	BCUT_SLOGP_1	Yes
47	0.77614	0.35532	0.29359	0.16393	BCUT_SLOGP_2	No
48	0.72364	0.30267	0.2664	0.03064	BCUT_SLOGP_3	No
49	0.78919	0.37096	0.36856	0.07489	BCUT_SMR_0	No
50	0.71194	0.29266	0.22733	0.15731	BCUT_SMR_1	No
51	0.76139	0.33903	0.30727	0.12927	BCUT_SMR_2	No
52	0.74679	0.32415	0.3191	0.00414	BCUT_SMR_3	No
53	0.79045	0.37253	0.33159	0.223	bpol	Yes
54	0.7489	0.25926	0.28193	0.07479	b_1rotN	No
55	0.70248	0.28494	0.20926	0.03375	b_1rotR	No
57	0.90949	0.28481	0.52067	0.23139	b_count	Yes
58	0.69412	0.27835	0.25373	0.291	b_double	Yes
59	0.83826	0.30149	0.40014	0.28016	b_heavy	Yes
60	0.67444	0.2637	0.20523	0.00888	b_max1len	No
61	0.77026	0.26357	0.30449	0.1559	b_rotN	No
62	0.71759	0.29743	0.26584	0.13141	b_rotR	No
63	0.89097	0.26222	0.45792	0.21207	b_single	Yes
67	0.77313	0.35188	0.35335	0.30093	chi0	Yes

#	Contingency coeff.	Cramér's V	Entropic Uncertainty	Linear R^2	Descriptor	Decision
68	0.80122	0.38654	0.37878	0.31759	chi0v	Yes
69	0.7784	0.35794	0.34557	0.25953	chi0v_C	Yes
70	0.76446	0.34232	0.31708	0.26328	chi0_C	Yes
71	0.73629	0.31412	0.2976	0.25823	chi1	Yes
72	0.77045	0.34887	0.33574	0.27264	chi1v	Yes
73	0.74716	0.32451	0.32002	0.25122	chi1v_C	Yes
74	0.72511	0.30396	0.30916	0.24935	chi1_C	Yes
79	0.75213	0.32946	0.31004	0.0051	dens	No
80	0.72409	0.30306	0.287	0.00459	density	No
81	0.79123	0.29684	0.38293	0.19691	diameter	No
86	0.66342	0.25595	0.22295	0.03612	E	No
88	0.75803	0.33551	0.327	0.16386	E_ele	No
89	0.75931	0.33684	0.28382	0.13445	E_nb	No
90	0.75706	0.3345	0.29178	0.03439	E_oop	No
95	0.79329	0.37612	0.31248	0.00515	E_sol	No
97	0.63791	0.23912	0.21431	0.09811	E_str	No
98	0.71099	0.29187	0.261	0.09433	E_strain	No
99	0.7754	0.35448	0.34419	0.08839	E_tor	No
100	0.76455	0.34241	0.2849	0.23431	E_vdw	Yes
108	0.72879	0.30724	0.26885	0.23606	GCUT_PEOE_0	Yes
109	0.71106	0.29193	0.23466	0.01643	GCUT_PEOE_1	No
110	0.7768	0.35609	0.27043	0.12631	GCUT_PEOE_2	No
111	0.78803	0.36951	0.33146	0.23193	GCUT_PEOE_3	Yes
113	0.70318	0.2855	0.22766	0.16233	GCUT_SLOGP_1	No
114	0.7267	0.30538	0.27005	0.18626	GCUT_SLOGP_2	No
115	0.77938	0.35909	0.33823	0.24391	GCUT_SLOGP_3	Yes
116	0.79455	0.37775	0.36045	0.06445	GCUT_SMR_0	No
117	0.71207	0.29277	0.23084	0.03826	GCUT_SMR_1	No
118	0.78667	0.36784	0.326	0.04582	GCUT_SMR_2	No
119	0.78048	0.36039	0.33189	0.23469	GCUT_SMR_3	Yes
120	0.72051	0.29994	0.2195	0.02008	glob	No
121	0.78386	0.36442	0.3611	0.22253	h_ema	Yes
122	0.81199	0.40159	0.35803	0.1117	h_emd	No
123	0.76473	0.34261	0.30946	0.18992	h_emd_C	No
124	0.76367	0.34146	0.29239	0.20665	h_logD	Yes
125	0.77756	0.35696	0.30073	0.21594	h_logP	Yes
126	0.80936	0.39781	0.35858	0.32336	h_logS	Yes
128	0.7392	0.31684	0.29671	0.26375	h_log_pbo	Yes
129	0.76545	0.34338	0.32111	0.28387	h_mr	Yes
135	0.79548	0.37895	0.3514	0.28532	Kier1	Yes
136	0.74019	0.31778	0.29021	0.19055	Kier2	No
137	0.80649	0.39376	0.3661	0.26369	Kier3	Yes
138	0.79852	0.38293	0.37956	0.31384	KierA1	Yes
139	0.7463	0.32367	0.30433	0.20783	KierA2	Yes
140	0.79112	0.37337	0.35759	0.27894	KierA3	Yes

#	Contingency coeff.	Cramér's V	Entropic Uncertainty	Linear R^2	Descriptor	Decision
141	0.76393	0.34174	0.25824	0.20898	KierFlex	Yes
142	0.77097	0.34946	0.31072	0.17618	lip_acc	No
146	0.82297	0.4182	0.35541	0.33057	logP(o/w)	Yes
147	0.80069	0.38582	0.34452	0.303	logS	Yes
149	0.81328	0.40348	0.38035	0.30291	MNDO_E	Yes
150	0.81698	0.40898	0.35177	0.26571	MNDO_Eele	Yes
151	0.74768	0.32503	0.29428	0.17347	MNDO_HF	No
152	0.72657	0.30526	0.23824	0.05427	MNDO_HOMO	No
153	0.71454	0.29485	0.22939	0.05427	MNDO_IP	No
155	0.75361	0.33097	0.31425	0.28057	mr	Yes
158	0.75409	0.33145	0.27931	0.00235	npr1	No
159	0.7322	0.31034	0.26987	0.00007	npr2	No
160	0.64823	0.22752	0.2554	0.02199	opr_brigid	No
163	0.75658	0.26544	0.30268	0.12194	opr_nrot	No
164	0.64709	0.24501	0.22701	0.17129	opr_violation	No
167	0.77215	0.35078	0.32007	0.1694	PEOE_PC+	No
168	0.75737	0.33482	0.31139	0.1694	PEOE_PC-	No
169	0.74689	0.32425	0.29318	0.02014	PEOE_RPC+	No
170	0.77546	0.35454	0.35688	0.2386	PEOE_RPC-	Yes
171	0.76762	0.34575	0.31059	0.17057	PEOE_VSA+0	No
172	0.73567	0.31354	0.27482	0.11071	PEOE_VSA+1	No
173	0.72329	0.30236	0.27538	0.11199	PEOE_VSA+2	No
176	0.64907	0.24631	0.22237	0.37385	PEOE_VSA+5	Yes
178	0.80146	0.38686	0.37998	0.26234	PEOE_VSA-0	Yes
179	0.71817	0.29793	0.25977	0.01867	PEOE_VSA-1	No
183	0.67339	0.26294	0.24094	0.30193	PEOE_VSA-5	Yes
184	0.75949	0.33703	0.27635	0.03108	PEOE_VSA-6	No
185	0.80449	0.391	0.30181	0.14166	PEOE_VSA_FHYD	No
186	0.78119	0.36122	0.32307	0.00575	PEOE_VSA_FNEG	No
187	0.79079	0.37295	0.34674	0.19011	PEOE_VSA_FPNEG	No
188	0.80704	0.39453	0.31123	0.14166	PEOE_VSA_FPOL	No
189	0.79334	0.3762	0.33215	0.00575	PEOE_VSA_FPOS	No
190	0.79641	0.38016	0.28139	0.08341	PEOE_VSA_FPPOS	No
191	0.76549	0.34343	0.29665	0.22032	PEOE_VSA_HYD	Yes
192	0.80021	0.38518	0.34784	0.38479	PEOE_VSA_NEG	Yes
193	0.79919	0.38383	0.36957	0.28054	PEOE_VSA_PNEG	Yes
194	0.8139	0.40439	0.35802	0.24361	PEOE_VSA_POL	Yes
195	0.775	0.35402	0.33603	0.12012	PEOE_VSA_POS	No
196	0.79084	0.37302	0.31549	0.17357	PEOE_VSA_PPOS	No
197	0.70034	0.28323	0.28582	0.00038	petitjean	No
198	0.73845	0.31614	0.31818	0.00105	petitjeanSC	No
200	0.80967	0.39826	0.37194	0.30978	PM3_E	Yes
201	0.80087	0.38606	0.34191	0.261	PM3_Eele	Yes
202	0.78899	0.3707	0.33726	0.28001	PM3_HF	Yes
203	0.68106	0.2685	0.22423	0.00004	PM3_HOMO	No

#	Contingency coeff.	Cramér's V	Entropic Uncertainty	Linear R^2	Descriptor	Decision
204	0.71527	0.29546	0.23452	0.00004	PM3_IP	No
205	0.79139	0.37372	0.33789	0.35832	PM3_LUMO	Yes
206	0.76859	0.34682	0.32835	0.29965	pmi	Yes
207	0.78498	0.36577	0.36936	0.25712	pmi1	Yes
208	0.76225	0.33994	0.32815	0.28971	pmi2	Yes
209	0.79852	0.38293	0.34363	0.29635	pmi3	Yes
210	0.81149	0.40087	0.38257	0.24743	pmiX	Yes
211	0.73542	0.31331	0.26299	0.09327	pmiY	No
212	0.83951	0.44602	0.38887	0.32992	pmiZ	Yes
304	0.81202	0.40164	0.37802	0.28996	Q_VSA_HYD	Yes
308	0.81202	0.40164	0.37802	0.28996	Q_VSA_POS	Yes
310	0.71743	0.29729	0.29411	0.20119	radius	Yes
312	0.78481	0.36557	0.35944	0.32043	rgyr	Yes
316	0.7745	0.35344	0.32169	0.18359	rsynth	No
317	0.79138	0.37369	0.31479	0.23819	SlogP	Yes
318	0.75007	0.32739	0.23233	0.01223	SlogP_VSA0	No
320	0.78776	0.36918	0.32962	0.36507	SlogP_VSA2	Yes
322	0.65447	0.24988	0.24607	0.01121	SlogP_VSA4	No
327	0.78668	0.36785	0.35622	0.29909	SlogP_VSA9	Yes
328	0.78232	0.36256	0.33996	0.28374	SMR	Yes
329	0.71622	0.29627	0.29568	0.3538	SMR_VSA0	Yes
330	0.74311	0.32058	0.26968	0.0002	SMR_VSA1	No
332	0.78448	0.36517	0.30415	0.11904	SMR_VSA3	No
335	0.76945	0.34776	0.33449	0.04564	SMR_VSA6	No
336	0.74855	0.32589	0.2649	0.21121	SMR_VSA7	Yes
337	0.76226	0.33996	0.32527	0.24128	std_dim1	Yes
338	0.7561	0.33351	0.31675	0.21048	std_dim2	Yes
339	0.81262	0.40251	0.35272	0.13501	std_dim3	No
340	0.7805	0.36041	0.34041	0.1597	TPSA	No
341	0.78579	0.36676	0.34637	0.31047	VAdjEq	Yes
342	0.78287	0.36323	0.35048	0.29577	VAdjMa	Yes
343	0.75061	0.32793	0.32411	0.25408	VDistEq	Yes
344	0.78309	0.36349	0.36067	0.30127	VDistMa	Yes
345	0.81202	0.40164	0.37802	0.28996	vdw_area	Yes
346	0.77538	0.35445	0.32933	0.27655	vdw_vol	Yes
347	0.76894	0.34721	0.32527	0.28111	vol	Yes
348	0.74251	0.32	0.31119	0.28159	VSA	Yes
349	0.67352	0.26304	0.22504	0.20761	vsa_acc	Yes
353	0.79638	0.38011	0.31941	0.24557	vsa_hyd	Yes
354	0.74589	0.32327	0.32659	0.31403	vsa_other	Yes
355	0.7809	0.36088	0.32071	0.12456	vsa_pol	No
356	0.77425	0.35316	0.32056	0.00154	vsurf_A	No
357	0.68974	0.27499	0.21223	0.05873	vsurf_CP	No
358	0.7929	0.37563	0.33488	0.23931	vsurf_CW1	Yes
359	0.76529	0.34321	0.29271	0.08084	vsurf_CW2	No

#	Contingency coeff.	Cramér's V	Entropic Uncertainty	Linear R^2	Descriptor	Decision
360	0.74712	0.32447	0.26188	0.02133	vsurf_CW3	No
361	0.71521	0.29541	0.24449	0.01185	vsurf_CW4	No
362	0.6983	0.28162	0.22844	0.00721	vsurf_CW5	No
366	0.77478	0.35376	0.29993	0.29927	vsurf_D1	Yes
367	0.78386	0.36441	0.30891	0.30239	vsurf_D2	Yes
368	0.80203	0.38763	0.31367	0.30026	vsurf_D3	Yes
369	0.82734	0.4252	0.35303	0.27628	vsurf_D4	Yes
370	0.77623	0.35543	0.28488	0.23811	vsurf_D5	Yes
371	0.76308	0.34084	0.27766	0.11491	vsurf_D6	No
372	0.68781	0.27353	0.23081	0.06904	vsurf_D7	No
373	0.76459	0.34246	0.32053	0.15316	vsurf_D8	No
374	0.74908	0.32641	0.20968	0.00215	vsurf_DD12	No
375	0.71212	0.29282	0.2143	0.02527	vsurf_DD13	No
376	0.69928	0.28239	0.22822	0.05385	vsurf_DD23	No
379	0.70078	0.28357	0.21544	0.02272	vsurf_DW23	No
381	0.69997	0.28293	0.22321	0.04913	vsurf_EDmin2	No
382	0.70554	0.2874	0.24136	0.05264	vsurf_EDmin3	No
383	0.73199	0.31015	0.23993	0.08894	vsurf_EWmin1	No
384	0.74246	0.31994	0.23273	0.08111	vsurf_EWmin2	No
385	0.75311	0.33045	0.23987	0.10871	vsurf_EWmin3	No
386	0.80534	0.39216	0.37562	0.33646	vsurf_G	Yes
387	0.79538	0.37882	0.36785	0.14505	vsurf_HB1	No
388	0.77233	0.35098	0.35316	0.1653	vsurf_HB2	No
389	0.7506	0.32792	0.30706	0.19516	vsurf_HB3	No
390	0.74996	0.32728	0.30663	0.1191	vsurf_HB4	No
391	0.70958	0.29071	0.24143	0.05666	vsurf_HB5	No
397	0.76376	0.34157	0.27924	0.081	vsurf_ID1	No
398	0.78629	0.36737	0.28913	0.08961	vsurf_ID2	No
399	0.82757	0.42557	0.33484	0.14876	vsurf_ID3	No
400	0.79498	0.3783	0.30541	0.16614	vsurf_ID4	No
401	0.7874	0.36873	0.28666	0.15443	vsurf_ID5	No
402	0.7906	0.37271	0.28115	0.00965	vsurf_ID6	No
403	0.69446	0.27861	0.24234	0.00556	vsurf_ID7	No
404	0.74213	0.31963	0.29096	0.01872	vsurf_ID8	No
405	0.71401	0.2944	0.25243	0.07175	vsurf_IW1	No
406	0.75551	0.3329	0.28444	0.00191	vsurf_IW2	No
407	0.7359	0.31375	0.28389	0.03454	vsurf_IW3	No
408	0.76685	0.34491	0.30913	0.02496	vsurf_IW4	No
409	0.76263	0.34035	0.3143	0.01156	vsurf_IW5	No
410	0.73195	0.31011	0.27726	0.00002	vsurf_IW6	No
411	0.72062	0.30003	0.24334	0.08157	vsurf_IW7	No
412	0.69494	0.27899	0.24608	0.05261	vsurf_IW8	No
413	0.77099	0.34948	0.30724	0.11241	vsurf_R	No
414	0.79746	0.38153	0.35303	0.31377	vsurf_S	Yes
415	0.78446	0.36514	0.3484	0.30032	vsurf_V	Yes

#	Contingency coeff.	Cramér's V	Entropic Uncertainty	Linear R^2	Descriptor	Decision
416	0.76597	0.34395	0.36344	0.27151	vsurf_W1	Yes
417	0.78717	0.36845	0.33961	0.18362	vsurf_W2	No
418	0.77258	0.35126	0.32497	0.15609	vsurf_W3	No
419	0.76054	0.33814	0.30791	0.08902	vsurf_W4	No
420	0.69904	0.2822	0.23026	0.04599	vsurf_W5	No
424	0.75723	0.33468	0.31818	0.30398	vsurf_Wp1	Yes
425	0.82599	0.42301	0.38437	0.16848	vsurf_Wp2	No
426	0.82086	0.41489	0.30323	0.01134	vsurf_Wp3	No
427	0.76619	0.34419	0.27452	0.02639	vsurf_Wp4	No
428	0.73778	0.31551	0.21752	0.03352	vsurf_Wp5	No
432	0.79647	0.38024	0.33862	0.31461	Weight	Yes
433	0.76733	0.34543	0.33355	0.21857	weinerPath	Yes
434	0.88475	0.32087	0.48244	0.27351	weinerPol	Yes
435	0.79935	0.38404	0.37815	0.31191	zagreb	Yes

Table S4. Equations describing models MLR1-MLR3.

Model	Equation
MLR1	17.0056 − 0.910189 × AM1_LUMO + 16.3448 × GCUT_PEOE_0 + 0.000489367 × VSA − 0.237012 × radius + 0.00037054 × vsa_hyd + 0.00396902 × vsurf_D3
MLR2	2.85947 + 0.798618 × SlogP + 0.14958 × b_double − 0.0482161 × b_single + 0.27467 × h_ema − 0.371147 × h_logP
MLR3	12.466 + 0.00809057 × ASA + 14.8266 × GCUT_PEOE_0 + 0.63541 × VDistEq − 0.0546541 × b_single + 0.190234 × chi1v_C − 0.371497 × radius

Table S5. Model performance assessment using different validation metrics.

Model	R^2	Q^2	R^2_{pred}	R^2_0	R'^2_0	R^2_p	$Q^2{}_{F2}$	$Q^2{}_{F3}$	CCC	MAE	σ_{MAE}	$\overline{R^2_m}$	ΔR^2_m
MLR1	0.731	0.648	0.708	0.715	0.522	0.722	0.705	0.614	0.817	0.507	0.207	0.625	0.048
MLR2	0.706	0.605	0.679	0.719	0.508	0.730	0.675	0.575	0.805	0.538	0.215	0.635	0.037
MLR3	0.716	0.626	0.671	0.691	0.432	0.701	0.667	0.564	0.790	0.490	0.246	0.598	0.163
RF1	0.918	0.510	0.784	0.785	0.556	0.834	0.783	0.745	0.856	0.462	0.148	0.751	0.136
RF2	0.812	0.462	0.788	0.786	0.669	0.793	0.785	0.802	0.870	0.443	0.116	0.687	0.169
RF3	0.897	0.538	0.710	0.596	0.236	0.598	0.576	0.759	0.729	0.491	0.137	0.483	0.021
SVM1	0.826	0.702	0.726	0.718	0.629	0.719	0.693	0.723	0.832	0.518	0.139	0.559	0.233
SVM2	0.891	0.604	0.717	0.726	0.421	0.761	0.713	0.625	0.808	0.530	0.186	0.674	0.076
SVM3	0.838	0.709	0.620	0.619	0.435	0.620	0.618	0.552	0.772	0.629	0.185	0.416	0.284
3D1	0.936	0.659	0.775	0.775	0.747	0.779	0.775	0.777	0.881	0.187	0.170	0.669	0.177
3D2	0.934	0.694	0.715	0.723	0.694	0.732	0.715	0.725	0.852	0.233	0.157	0.635	0.101
3D3	0.923	0.644	0.765	0.757	0.700	0.758	0.765	0.773	0.865	0.212	0.143	0.669	0.077
3D4	0.932	0.624	0.793	0.801	0.828	0.828	0.793	0.822	0.905	0.187	0.134	0.658	0.164
3D5	0.909	0.618	0.841	0.826	0.796	0.826	0.841	0.816	0.906	0.203	0.099	0.761	0.048
CoefVar * (%)	10.3	12.7	7.0	8.3	27.5	9.2	9.3	13.6	5.5	33.0	22.3	15.0	64.3

* CoefVar = Coefficient of Variation.

Table S6. Definition of validation parameters used for assessing the performance of the models.

Parameter	Mathematical Definition	Reference
1	$R^2 = 1 - \frac{\sum(Y_{obs} - Y_{pred})^2}{\sum(Y_{obs} - \overline{Y_{obs}})^2}$	[64]
2	$Q^2 = 1 - \frac{\sum(Y_{obs} - Y_{pred(LOO)})^2}{\sum(Y_{obs} - \overline{Y_{obs}})^2}$	[64]
3	$R^2_{pred} = 1 - \frac{\sum(Y_{obs(test)} - Y_{pred(test)})^2}{\sum(Y_{obs(test)} - \overline{Y_{train}})^2}$	[64]
4	$R^2_p = \frac{\sum(Y_{obs} - \overline{Y_{obs}})(Y_{pred} - \overline{Y_{pred}})}{\sqrt{\{[\sum(Y_{obs} - \overline{Y_{obs}})][\sum(Y_{pred} - \overline{Y_{pred}})]\}^2}}$	[35]
5	$k = \frac{\sum Y_{obs} Y_{pred}}{\sum Y^2_{pred}}$	[35]
6	$k' = \frac{\sum Y_{obs} Y_{pred}}{\sum Y^2_{obs}}$	[35]
7 *	$R^2_0 = 1 - \frac{\sum(Y_{pred} - kY_{pred})^2}{\sum(Y_{pred} - \overline{Y_{pred}})^2}$	[35]
8 *	$R'^2_0 = 1 - \frac{\sum(Y_{obs} - k'Y_{obs})^2}{\sum(Y_{obs} - \overline{Y_{obs}})^2}$	[35]
9	$R^2_m = R^2_p \times \left(1 - \sqrt{(R^2_p - R^2_0)}\right)$	[57,65,66]
10	$R'^2_m = R^2_p \times \left(1 - \sqrt{(R^2_p - R'^2_0)}\right)$	[57,65,66]
11	$\overline{R^2_m} = \frac{R^2_m + R'^2_m}{2}$	[57,65,66]
12	$\Delta R^2_m = \lvert R^2_m - R'^2_m \rvert$	[57,65,66]
13	${Q^2}_{F2} = 1 - \frac{\sum(Y_{obs(test)} - Y_{pred(test)})^2}{\sum(Y_{obs(test)} - \overline{Y_{test}})^2}$	[60]

Parameter	Mathematical Definition	Reference
14	$Q^2_{F3} = 1 - \frac{\left[\sum\left(Y_{obs(test)} - Y_{pred(test)}\right)^2\right]/N_{test}}{\left[\sum\left(Y_{obs(test)} - \overline{Y_{train}}\right)^2\right]/N_{train}}$	[61]
15	$CCC = \frac{2\sum(Y_{obs} - \overline{Y_{obs}})\left(Y_{pred} - \overline{Y_{pred}}\right)}{\sum(Y_{obs} - \overline{Y_{obs}})^2 + \sum\left(Y_{pred} - \overline{Y_{pred}}\right)^2 + N_{test}\left(\overline{Y_{obs}} - \overline{Y_{pred}}\right)^2}$	[62,67]
16	$MAE = \frac{\sum\left\|Y_{obs(test)} - Y_{pred(test)}\right\|}{N_{test}}$	[58]
17	$Scaled\ Y_i = \frac{Y_i - Y_{min}}{Y_{max} - Y_{min}}$	[57]

* Scaled Y_i must be used (Equation (17)).

Table S7. Definition of consensus scoring functions [37].

Scoring Function	Parameters [a]		
	$P1$	$P2$	$P3$ [b]
$F1=\sum_{i=1}^{7}P1_i+\sum_{i=1}^{3}P2_i+\sum_{i=1}^{2}P3_i$	(1) $R^2>0.8$<br>(2) $Q^2>0.5$<br>(3) $R^2_{pred}>0.6$<br>(4) $Q^2_{F2}>0.5$<br>(5) $Q^2_{F3}>0.5$<br>(6) $CCC>0.85$<br>(7) $\overline{R^2_m}>0.5$	(1) $\frac{R^2-R_0^2}{R^2}\ or\ \frac{R^2-R_0'^2}{R^2}<0.1$<br>(2) $\lvert R_0^2-R_0'^2\rvert<0.3$<br>(3) $\Delta R_m^2<0.2$	(1) $MAE\leq 0.34\ and\ MAE+3\sigma_{MAE}\leq 0.68$<br>(2) $MAE\leq 0.51\ and\ MAE+3\sigma_{MAE}\leq 0.85$
$F2=\sum_{i=1}^{8}P1_i+\sum_{i=1}^{3}P2_i+\sum_{i=1}^{2}P3_i$	(1) $R^2-0.8$<br>(2) $Q^2-0.5$<br>(3) $R^2_{pred}-0.6$<br>(4) $Q^2_{F2}-0.5$<br>(5) $Q^2_{F3}-0.5$<br>(6) $CCC-0.85$<br>(7) $\overline{R^2_m}-0.5$<br>(8) $R^2-Q^2_{F2}$<br>$if\ R^2>Q^2_{F2}$<br>$or\ -Q^2_{F2}$<br>$if\ R^2<Q^2_{F2}$	(1) $if\ \frac{R^2-R_0^2}{R^2}<\frac{R^2-R_0'^2}{R^2}$:<br>$0.1-\frac{R^2-R_0^2}{R^2}$<br>$otherwise: 0.1-\frac{R^2-R_0'^2}{R^2}$<br>(2) $0.3-\lvert R_0^2-R_0'^2\rvert$<br>(3) $0.2-\Delta R_m^2$	(1) $If\ MAE-0.51>0\ or$<br>$MAE+3\sigma_{MAE}-0.85>0$:<br>$MAE-0.51$<br>$if\ MAE-0.51>MAE+3\sigma_{MAE}-0.85$<br>and<br>$3\sigma_{MAE}-0.85$<br>$if\ MAE-0.51<MAE+3\sigma_{MAE}-0.85$<br>(2) $If\ MAE-0.34>0\ and$<br>$MAE+3\sigma_{MAE}-0.68>0$<br>$Or\ if\ MAE-0.34<0\ and$<br>$MAE+3\sigma_{MAE}-0.68<0$:<br>$(0.34-MAE)+(0.68-[MAE+3\sigma_{MAE}])$

[a] All the definitions of the involved parameters are found in the previous table (Table S4). [b] Thresholds used as originally reported (see [37] for details).

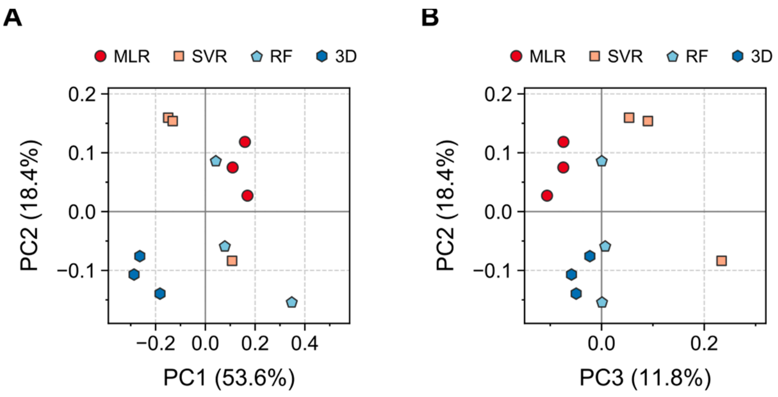

Figure S1. Comparison of models' performance using different metrics and PCA. (**A**) PC1-PC2 and (**B**) PC3-PC2 score plots from PCA on validation metrics.

Table S8. Activity predictions by model 3D4.

ID	pIC50 Exp.*	pIC50 Pred.	Set	ID	pIC50 Exp.*	pIC50 Pred.	Set
**1**	5.11	5.06	training	**36**	4.66	4.46	training
**2**	5.13	5.31	test	**37**	4.52	4.47	training
**3**	5.38	5.35	test	**38**	4.76	4.78	training
**4**	4.97	5.26	test	**39**	4.73	4.39	test
**5**	5.22	5.44	test	**40**	4.86	4.73	training
**6**	4.97	5.09	training	**41**	4.61	4.85	training
**7**	5.04	5.46	test	**42**	4.52	4.51	test
**8**	5.40	5.53	test	**43**	4.50	4.60	training
**9**	5.51	5.41	test	**44**	4.47	4.19	training
**10**	5.68	5.84	training	**45**	4.79	4.77	test
**11**	4.90	5.57	training	**46**	4.82	5.16	training
**12**	5.41	5.34	training	**47**	4.97	4.80	training
**13**	6.27	5.49	training	**48**	4.53	4.80	training
**14**	5.78	5.40	training	**49**	4.94	5.06	training
**15**	5.78	5.73	test	**50**	4.87	4.65	training
**16**	4.63	5.28	training	**51**	4.66	4.61	test
**17**	5.20	5.44	test	**52**	4.76	4.96	test
**18**	5.71	5.42	training	**53**	4.92	4.72	test
**19**	5.19	4.74	training	**54**	4.99	4.45	training
**20**	3.80	4.42	training	**55**	4.36	4.20	test
**21**	5.05	4.50	training	**56**	4.87	4.46	test
**22**	4.27	4.55	training	**57**	5.40	4.89	training
**23**	4.08	4.45	training	**58**	4.97	4.88	training
**24**	3.67	3.87	training	**59**	4.77	5.23	training
**25**	5.42	5.37	training	**60**	4.75	4.63	training
**26**	5.35	5.43	training	**61**	4.49	4.58	test
**27**	5.47	5.71	training	**62**	5.25	4.87	training
**28**	5.16	5.18	training	**63**	5.69	5.22	training
**29**	4.23	4.37	training	**64**	4.88	5.47	training
**30**	3.73	4.17	test	**65**	4.89	5.37	training
**31**	4.00	4.12	test	**66**	5.20	5.19	training
**32**	3.87	4.05	training	**67**	4.78	4.59	training
**33**	3.80	4.11	training	**68**	4.42	4.27	training
**34**	4.15	3.92	test	**69**	4.73	4.67	training
**35**	4.23	4.14	training	**70**	4.79	4.72	training

* Taken from [26].

Table S9. Molecular descriptors considered for model building.

Molecular Descriptor	Description
AM1_E	The total SCF energy (kcal/mol)
AM1_Eele	The electronic energy (kcal/mol)
AM1_HF	The heat of formation (kcal/mol)
AM1_HOMO	HOMO energy (eV)
apol	Sum of the atomic polarizabilities
ASA	Water accessible surface area
ASA_H	Water accessible surface area of all hydrophobic atoms
ast_violation	Astex fragment-like violation count
ast_violation_ext	Astex fragment-like violation count (extended)
a_count	Number of atoms (including implicit hydrogens)
a_heavy	Number of heavy atoms
a_hyd	Number of hydrophobic atoms
a_IC	Atom information content (total)
a_nC	Number of carbon atoms
a_nO	Number of oxygen atoms
BCUT_SLOGP_1	2nd BCUT descriptor using atomic contribution to LogP
bpol	Sum of the absolute value of the difference between atomic polarizabilities of all bonded atoms in the molecule
b_count	Number of bonds (including implicit hydrogens)
b_double	Number of double bonds. Aromatic bonds are not considered to be double bonds.
b_heavy	Number of bonds between heavy atoms
b_single	Number of single bonds
chi0	Atomic connectivity index (order 0)
chi0v	Atomic valence connectivity index (order 0)
chi0v_C	Carbon valence connectivity index (order 0)
chi0_C	Carbon connectivity index (order 0)
chi1	Atomic connectivity index (order 1)
chi1v	Atomic valence connectivity index (order 1)
chi1v_C	Carbon valence connectivity index (order 1)
chi1_C	Carbon connectivity index (order 1)
E_vdw	van der Waals component of the potential energy
GCUT_PEOE_0	Smallest GCUT descriptor using PEOE partial charges
GCUT_PEOE_3	Largest GCUT descriptor using PEOE partial charges
GCUT_SLOGP_3	Largest GCUT descriptor using atomic contribution to LogP
GCUT_SMR_3	Largest GCUT descriptor using atomic contribution to molar refractivity
h_ema	Sum of hydrogen bond acceptor strengths
h_logD	The octanol/water distribution coefficient at pH 7
h_logP	Log of the octanol/water partition coefficient using an 8-parameter model based on Hückel Theory
h_logS	Log of the aqueous solubility (mol/L) using a 7-parameter model based on Hückel Theory
h_log_pbo	Sum of log (1 + pi bond order) for all bonds

Molecular Descriptor	Description
h_mr	Molar refractivity using a 4-parameter model based on Hückel Theory
Kier1	1st kappa shape index
Kier3	3rd kappa shape index
KierA1	1st alpha modified shape index
KierA2	2nd alpha modified shape index
KierA3	3rd alpha modified shape index
KierFlex	Kier molecular flexibility index
logP(o/w)	Log octanol/water partition coefficient
logS	Log of the aqueous solubility (mol/L)
MNDO_E	The total SCF energy (kcal/mol)
MNDO_Eele	The electronic energy (kcal/mol)
mr	Molecular refractivity
PEOE_RPC-	Relative negative partial charge using PEOE method
PEOE_VSA+5	Sum of van der Waals surface area where partial charge is in the range [0.25, 0.30)
PEOE_VSA-0	Sum of van der Waals surface area where partial charge is in the range [-0.05, 0.00)
PEOE_VSA-5	Sum of van der Waals surface area where partial charge is in the range [-0.30, -0.25)
PEOE_VSA_HYD	Total hydrophobic van der Waals surface area
PEOE_VSA_NEG	Total negative van der Waals surface area
PEOE_VSA_PNEG	Total negative polar van der Waals surface area
PEOE_VSA_POL	Total polar van der Waals surface area
PM3_E	The total SCF energy (kcal/mol)
PM3_Eele	The electronic energy (kcal/mol)
PM3_HF	The heat of formation (kcal/mol)
PM3_LUMO	LUMO energy (eV)
pmi	Principal moment of inertia
pmi1	1st diagonal element of diagonalized moment of inertia tensor
pmi2	2nd diagonal element of diagonalized moment of inertia tensor
pmi3	3rd diagonal element of diagonalized moment of inertia tensor
pmiX	*x* component of the principal moment of inertia
pmiZ	*z* component of the principal moment of inertia
Q_VSA_HYD	Total hydrophobic van der Waals surface area
Q_VSA_POS	Total positive van der Waals surface area
radius	Smallest entry in the distance matrix
rgyr	Radius of gyration
SlogP	Log of the octanol/water partition coefficient
SlogP_VSA2	Sum of van der Waals surface area where the contribution to SlogP is in the range (-0.20, 0.00]
SlogP_VSA9	Sum of van der Waals surface area where the contribution to SlogP is > 0.40
SMR	Molecular refractivity
SMR_VSA0	Sum of van der Waals surface area where the contribution to SMR is in the range (0.00, 0.11]
SMR_VSA7	Sum of van der Waals surface area where the contribution to SMR is > 0.56

Molecular Descriptor	Description
std_dim1	Standard dimension 1
std_dim2	Standard dimension 2
VAdjEq	Vertex adjacency information (equality)
VAdjMa	Vertex adjacency information (magnitude)
VDistEq	If *m* is the sum of the distance matrix entries then VdistEq is defined to be the sum of log2 *m* - *pi* log2 *pi* / m where pi is the number of distance matrix entries equal to i.
VDistMa	If *m* is the sum of the distance matrix entries then VDistMa is defined to be the sum of log2 *m* - *Dij* log2 *Dij* / *m* over all *i* and *j*
vdw_area	Area of van der Waals surface ($Å^2$)
vdw_vol	van der Waals volume ($Å^3$)
vol	Van der Waals volume
VSA	Van der Waals surface area
vsa_acc	Approximation to the sum of VDW surface areas ($Å^2$) of pure hydrogen bond acceptors
vsa_hyd	Approximation to the sum of VDW surface areas of hydrophobic atoms ($Å^2$)
vsa_other	Approximation to the sum of VDW surface areas of atoms typed as "other" ($Å^2$)
vsurf_CW1	Capacity factor at -0.2
vsurf_D1	Hydrophobic volume at -0.2
vsurf_D2	Hydrophobic volume at -0.4
vsurf_D3	Hydrophobic volume at -0.6
vsurf_D4	Hydrophobic volume at -0.8
vsurf_D5	Hydrophobic volume at -1.0
vsurf_G	Surface globularity
vsurf_S	Interaction field area
vsurf_V	Interaction field volume
vsurf_W1	Hydrophilic volume at -0.2
vsurf_Wp1	Polar volume at -0.2
Weight	Molecular weight (CRC)
wienerPath	Wiener path number
wienerPol	Wiener polarity number
zagreb	Zagreb index
